# Cognitive function during exercise under severe hypoxia

**DOI:** 10.1038/s41598-017-10332-y

**Published:** 2017-08-30

**Authors:** Takaaki Komiyama, Keisho Katayama, Mizuki Sudo, Koji Ishida, Yasuki Higaki, Soichi Ando

**Affiliations:** 1 Graduate School of Sports and Health Science, Fukuoka University, Fukuoka, 814-0180 Japan; 2 Research Center of Health, Physical Fitness and Sports, Nagoya University, 464-8601 Nagoya, Japan; 3 Meiji Yasuda Life Foundation of Health and Welfare, 192-0001 Tokyo, Japan; 4 Faculty of Sports Science, Fukuoka University, Fukuoka, 814-0180 Japan; 5 Graduate School of Informatics and Engineering, The University of Electro-Communications, Tokyo, 182-8585 Japan

## Abstract

Acute exercise has been demonstrated to improve cognitive function. In contrast, severe hypoxia can impair cognitive function. Hence, cognitive function during exercise under severe hypoxia may be determined by the balance between the beneficial effects of exercise and the detrimental effects of severe hypoxia. However, the physiological factors that determine cognitive function during exercise under hypoxia remain unclear. Here, we examined the combined effects of acute exercise and severe hypoxia on cognitive function and identified physiological factors that determine cognitive function during exercise under severe hypoxia. The participants completed cognitive tasks at rest and during moderate exercise under either normoxic or severe hypoxic conditions. Peripheral oxygen saturation, cerebral oxygenation, and middle cerebral artery velocity were continuously monitored. Cerebral oxygen delivery was calculated as the product of estimated arterial oxygen content and cerebral blood flow. On average, cognitive performance improved during exercise under both normoxia and hypoxia, without sacrificing accuracy. However, under hypoxia, cognitive improvements were attenuated for individuals exhibiting a greater decrease in peripheral oxygen saturation. Cognitive performance was not associated with other physiological parameters. Taken together, the present results suggest that arterial desaturation attenuates cognitive improvements during exercise under hypoxia.

## Introduction

Sufficient oxygen delivery and perfusion to brain tissue is critical for avoiding the life-threatening consequences of hypoxic environments^1^. Under hypoxic conditions, increases in cerebral blood flow serve to maintain oxygen delivery to the brain at rest^2–4^. Nevertheless, hypoxia may have detrimental effects on the central nervous system and brain function^1, 5, 6^. Indeed, a growing body of literature suggests that cognitive function is impaired under hypoxia^7–9^. Importantly, these impairments appear to be exaggerated as the severity of hypoxia increases^7–9^. This notion is in line with a recent meta-analytic review demonstrating that arterial oxygen partial pressure (PaO_2_) is the key predictor of cognitive function under hypoxia^10^.

In contrast, acute exercise appears to improve cognitive function^11, 12^. It has been suggested that increases in arousal to an optimal level lead to improvements in cognitive function during exercise^11, 13^. Although the physiological mechanisms underlying these improvements are still unclear, the noradrenergic and dopaminergic systems may be involved in cognitive improvement^14–16^. As mentioned above, hypoxia may have detrimental effects on cognitive function. Thus, cognitive function during exercise under severe hypoxia may be determined by the balance between the beneficial effects of acute exercise and the detrimental effects of hypoxia.

Recent studies have examined the combined effects of exercise and hypoxia on cognitive function^17–21^. Some researchers have reported that moderate exercise improves cognitive function under moderate to severe hypoxia^17, 19, 21^, while others found that cognitive function was impaired during moderate exercise under hypoxia^18, 20^. Thus, the combined effects of moderate exercise and hypoxia on cognitive function are still controversial in the literature. The discrepancies between previous studies may be related to differences in experimental conditions. Therefore, identifying the physiological factors that affect cognitive function during exercise under hypoxia may provide valuable insight.

Under hypoxia, PaO_2_ and peripheral oxygen saturation (SpO_2_) progressively decreases as the severity of hypoxia increases^2, 4^. Brain desaturation and resultant biological processes have been suggested to impair cognitive function under hypoxia, and these impairments are reported to worsen as the severity of hypoxia increases^7–9^. Since acute exercise under hypoxia induces progressive brain desaturation^22, 23^, cognitive improvements may be attenuated as brain desaturation proceeds during exercise under hypoxia. Thus, we hypothesised that decreases in SpO_2_ and cerebral oxygenation may attenuate cognitive improvements during moderate exercise under severe hypoxia.

Increased cerebral blood flow during exercise contributes to increase in cerebral oxygen delivery in response to cerebral oxygen metabolism^3^. However, despite increases in cerebral blood flow, cerebral oxygen delivery has been found to decrease during exercise under severe hypoxia due to progressive brain desaturation^22, 23^. Hence, the magnitude of cerebral blood flow increases and alterations in cerebral oxygen delivery may be critical for meeting the metabolic demands during exercise under severe hypoxia. We examined whether alterations in middle cerebral artery mean velocity (MCA V_mean_) and/or cerebral oxygen delivery are related to cognitive function during exercise under severe hypoxia.

**Figure 1:**
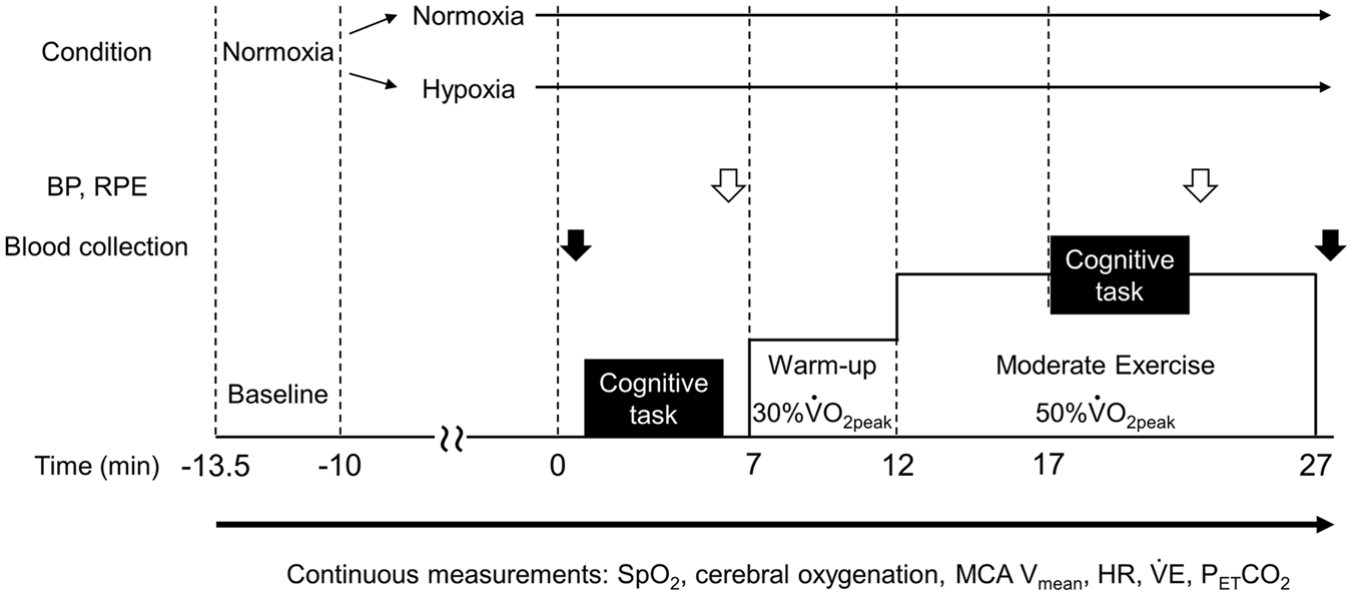
Experimental protocol. The white arrows indicate the timing of BP and RPE measurements. The black arrows indicate the timing of blood collection. BP, blood pressure; RPE, ratings of perceived exertion; SpO_2_, peripheral oxygen saturation; MCA V_mean_, middle cerebral artery mean velocity; HR, heart rate; ${\dot{{\rm{V}}}}_{{\rm{E}}}$ minute ventilation; P_ET_CO_2_, end-tidal partial pressure of carbon dioxide.

The purpose of the current study was to identify the physiological factors that determine cognitive function during exercise under severe hypoxia. We assessed cognitive function at rest and during exercise under either normxia or hypoxia (Fig. 1). We also focused on the ways that alterations in SpO_2_, cerebral oxygenation, cerebral blood flow, and cerebral oxygen delivery affect cognitive function during exercise under severe hypoxia. The findings from the present study will extend prior knowledge about the interactions between exercise and cognition under hypoxia, which has implications for sports, work, and recreational activities at high altitude.

## Results

### Cardiorespiratory parameters

Peak oxygen uptake $({\dot{{\rm{V}}}{\rm{O}}}_{{\rm{2peak}}})$ at exhaustion during maximal exercise was greater under normoxia (3.11 ± 0.27 mL/min) than that under hypoxia (2.23 ± 0.22 mL/min) (t_12_ = 9.76, p < 0.001). Absolute workload at 50% ${\dot{{\rm{V}}}{\rm{O}}}_{{\rm{2peak}}}$ was higher under normoxia (101.8 ± 13.7 W) than hypoxia (76.1 ± 9.1 W) (t_12_ = 7.23, p < 0.001). Nevertheless, actual percentage of ${\dot{{\rm{V}}}{\rm{O}}}_{{\rm{2peak}}}$ during exercise at 50% ${\dot{{\rm{V}}}{\rm{O}}}_{{\rm{2peak}}}$ was not different between under normoxia (48.2 ± 3.0%) and hypoxia (49.7 ± 5.1%) (t_12_ = −1.01, p = 0.33).

### Cognitive tasks

**Figure 2:**
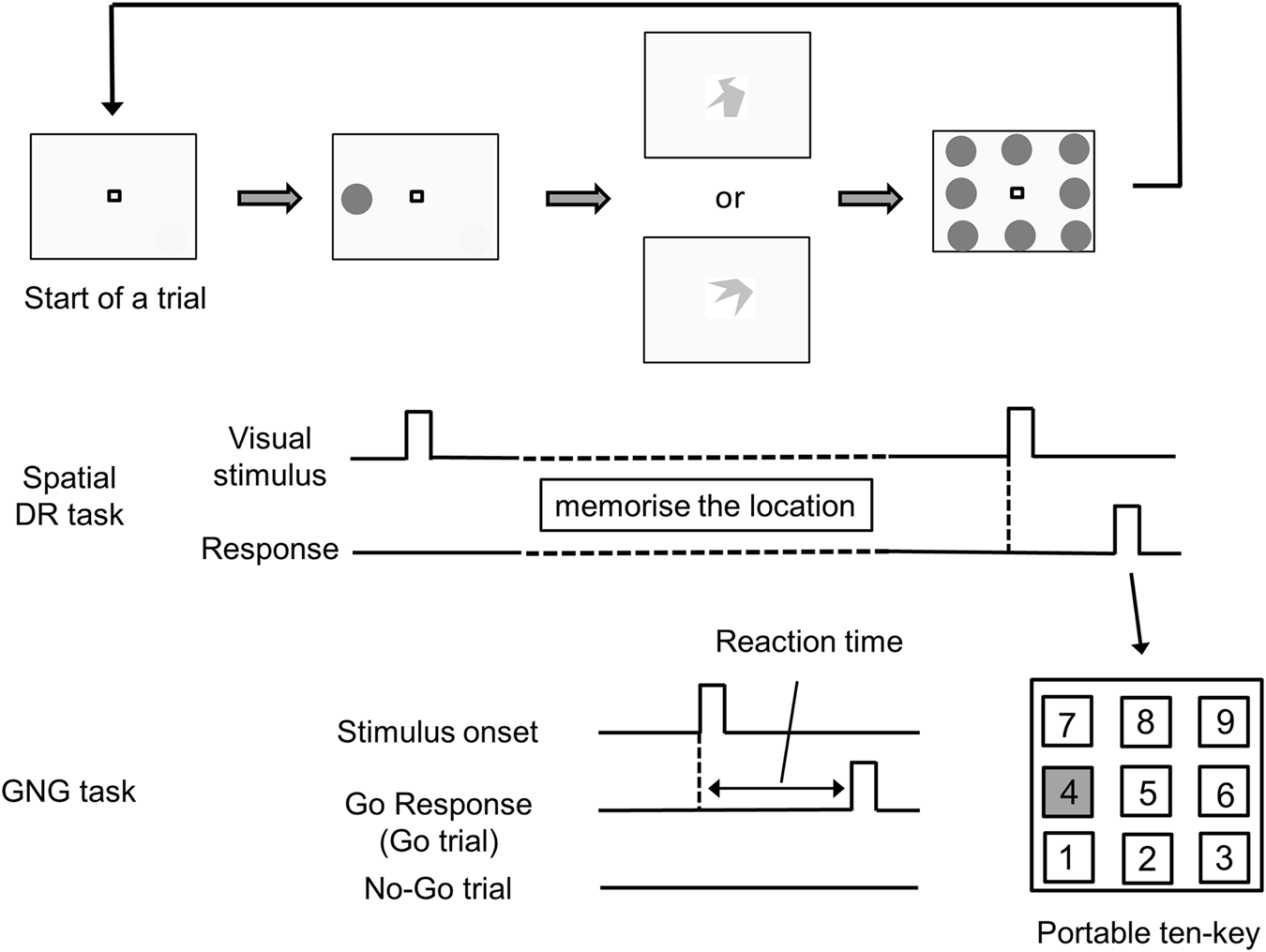
Schematic figure of the present cognitive tasks. The cognitive tasks included the spatial delayed-response (DR) task and the Go/No-Go (GNG) task. The cognitive tasks started with the spatial DR task. At the beginning of the spatial DR task, a visual cue was presented at one of the eight locations. The participants were asked to memorise the location. Then, the participants performed the GNG task. In the GNG task, one of the paired figures was randomly presented. When the presented figure was the target (“Go trial”), participants released a shift key as quickly as possible. If the figure was not the target (“No-Go trial”), participants continued holding the shift key down. After a trial in the GNG task, visual stimuli were presented at eight locations surrounding the fixation point. The participants responded by pressing the button of the ten-key corresponding to the memorised location in the preceding spatial DR task. In this case, participants pressed the number 4. The sequence was defined as a trial.

**Figure 3:**
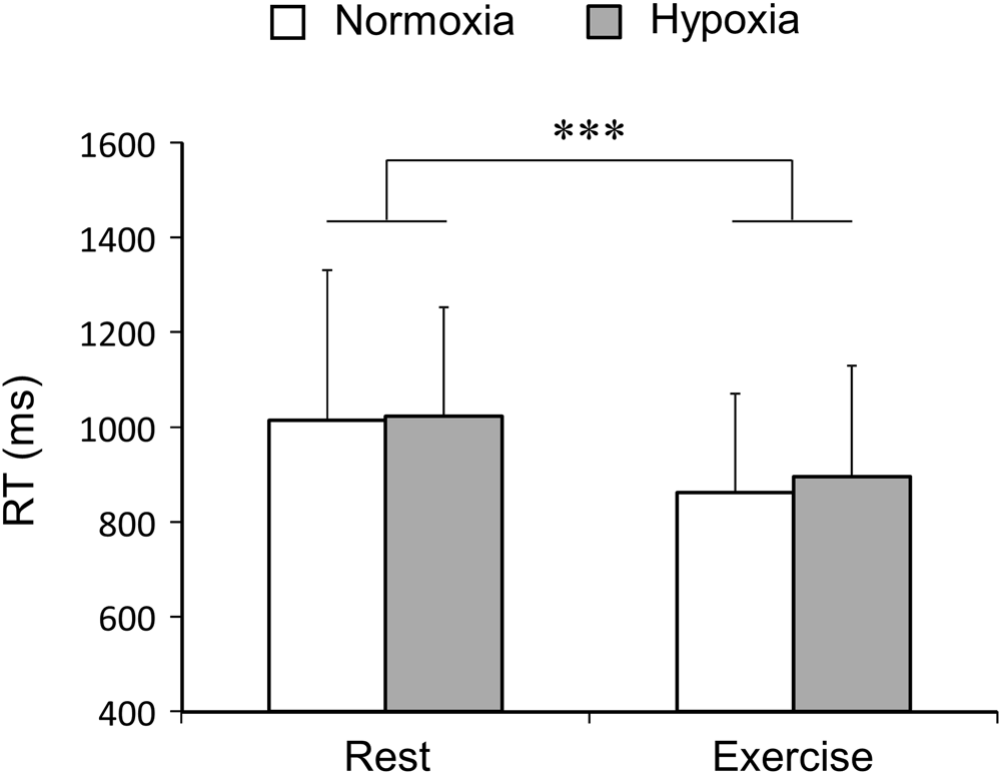
Reaction time (RT) on Go trials in the Go/No-Go task. ***p < 0.001 significant main effect of exercise (rest vs. exercise).

Figure 2 shows schematic figure of the spatial delay-response (DR) and Go/No-Go (GNG) tasks (See details in Cognitive tasks in Materials and methods). The present cognitive task started with the spatial DR task, followed by the GNG task. Figure 3 shows reaction time (RT) in the Go trial of the GNG task. At rest and during exercise, we observed no differences in RT between normoxia and hypoxia (F_1,12_ = 0.15, *p* = 0.71). In contrast, RT decreased during exercise compared with rest (F_1,12_ = 29.52, *p* < 0.001). The magnitude of the decrease in RT was no different between conditions (F_1,12_ = 0.16, *p* = 0.69). Response time in the spatial DR task was not different at rest and during exercise under normoxia and hypoxia (rest under normoxia: 450 ± 117 ms, exercise under normoxia: 474 ± 135 ms, rest under hypoxia: 477 ± 115 ms, exercise under hypoxia: 440 ± 97 ms, all *p* > 0.15). The accuracy of cognitive performance was relatively high throughout the experiment (Table 1). Neither hypoxia nor exercise affected the accuracy of the GNG and the spatial DR tasks (all *p* > 0.14). RT in the first Go trial after trials were reversed or a new pair of figures appeared was not different from mean RT at rest and during exercise under normoxia and hypoxia (all *p* > 0.20), which suggests that the participants adapted to new relationships immediately in the present study.

**Table 1:** Cognitive task accuracy and physiological parameters at rest and during exercise under normoxia and hypoxia.

Condition	Variable	Rest	Exercise	After
Normoxia	Accuracy of the Spatial DR task, %	99.7 ± 1.1	99.1 ± 1.7	
	Accuracy of the GNG task: Go trial, %	95.4 ± 7.1	95.6 ± 7.5	
	Accuracy of the GNG task: No-Go trial, %	89.9 ± 8.7	92.1 ± 8.0	
	HR, beats/min	69.5 ± 9.5	124.8 ± 9.2^***^	
	RPE	8.3 ± 1.5	12.8 ± 1.5^***^	
	SBP, mmHg	126.6 ± 10.8	175.7 ± 13.7^***^	
	DBP, mmHg	82.4 ± 6.9	84.3 ± 14.7	
	${\dot{{\rm{V}}}}_{{\rm{E}}}$, L/min	9.7 ± 2.1	41.9 ± 3.3^***^	
	P_ET_CO_2_, mmHg	30.9 ± 2.8	41.0 ± 2.7^###^	
	Blood lactate, mmol/L	1.1 ± 0.2		1.4 ± 0.4
	Blood glucose, mg/dL	88.4 ± 8.7		74.0 ± 8.6^***^
Hypoxia	Accuracy of the Spatial DR task, %	97.9 ± 3.3	98.4 ± 2.5	
	Accuracy of the GNG task: Go trial, %	93.6 ± 6.7	93.6 ± 9.6	
	Accuracy of the GNG task: No-Go trial, %	89.3 ± 10.5	90.6 ± 9.0	
	HR, beats/min	79.4 ± 10.2^††^	131.5 ± 15.1^***, ††^	
	RPE	7.8 ± 1.7	11.8 ± 1.2^***^	
	SBP, mmHg	122.5 ± 12.2	173.8 ± 18.4^***^	
	DBP, mmHg	83.6 ± 10.5	78.8 ± 13.1	
	${\dot{{\rm{V}}}}_{{\rm{E}}}$, L/min	9.5 ± 1.5	39.9 ± 5.7^***^	
	P_ET_CO_2_, mmHg	32.7 ± 3.2^‡^	36.1 ± 2.4^##, ‡‡‡^	
	Blood lactate, mmol/L	1.2 ± 0.3		1.9 ± 0.7^##, ‡‡‡^

Values are mean ± SD. ***p < 0.001 vs. rest (main effect of exercise), ^##^p < 0.01, ^###^p < 0.001 vs. rest, ^††^p < 0.01 vs. normoxia (main effect of condition), ^‡^p < 0.05, ^‡‡‡^p < 0.001 vs. normoxia. HR, heart rate; RPE, ratings of perceived exertion; SBP, systolic blood pressure; DBP, diastolic blood pressure; ${\dot{{\rm{V}}}}_{{\rm{E}}}$, minute ventilation; P_ET_CO2, end-tidal partial pressure of carbon dioxide.

### Physiological parameters

The results of physiological parameters are shown in Table 1. Heart rate (HR) was greater under hypoxia compared with normoxia (F_1,12_ = 14.06, *p* = 0.003). HR increased during exercise relative to rest (F_1,12_ = 345.94, *p* < 0.001). Ratings of perceived exertion (RPE) increased during exercise relative to rest (F_1,12_ = 114.87, *p* < 0.001). RPE was no different between normoxia and hypoxia (F_1,12_ = 2.79, *p* = 0.12). Systolic blood pressure (SBP) increased during exercise relative to rest (F_1,12_ = 322.33, *p* < 0.001), while diastolic blood pressure (DBP) did not change during exercise (F_1,12_ = 0.29, *p* = 0.60). Hypoxia failed to influence SBP (F_1,12_ = 1.24, *p* = 0.29) and DBP (F_1,12_ = 0.92, *p = *0.36). Minute ventilation (${\dot{{\rm{V}}}}_{{\rm{E}}}$) increased during exercise relative to rest (F_1,12_ = 1499.56, *p* < 0.001). The degree of increase in ${\dot{{\rm{V}}}}_{{\rm{E}}}$ was no different between normoxia and hypoxia (F_1,12_ = 1.08, *p* = 0.32). Alterations in end-tidal partial pressure of CO_2_ (P_ET_CO_2_) were significantly different between normoxia and hypoxia (F_1,12_ = 37.57, *p* < 0.001). P_ET_CO_2_ increased during exercise under normoxia (t_12_ = −9.47, *p* < 0.001) and hypoxia (t_12_ = −3.37, *p* < 0.01) compared with rest. During exercise, P_ET_CO_2_ was lower under hypoxia compared with normoxia (t_12_ = 6.17, *p* < 0.001). Increases in blood lactate level after exercise differed between normoxia and hypoxia (F_1,12_ = 8.79, *p* = 0.012). Although the blood lactate level increased after exercise under hypoxia (t_12_ = −3.09, *p* < 0.01), it did not increase significantly under normoxia (t_12_ = −1.85, *p* = 0.09). After exercise, the blood lactate level was higher under hypoxia than normoxia (t_12_ = −4.65, *p* < 0.001). The blood glucose level decreased after exercise (F_1,12_ = 28.46, *p* < 0.001).

### SpO_2_, cerebral oxygenation, cerebral blood flow, and cerebral oxygen delivery

**Figure 4:**
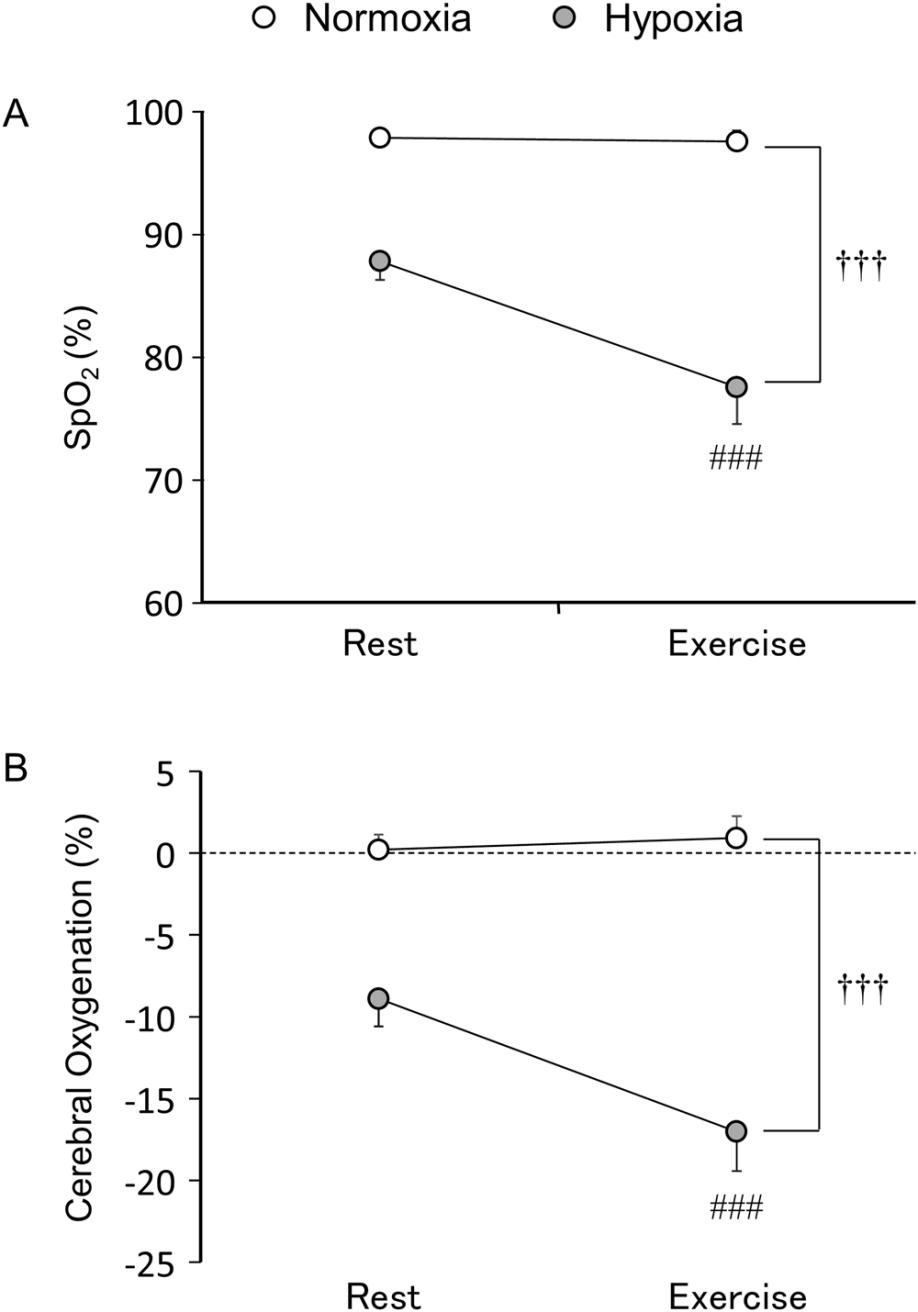
Peripheral oxygen saturation (SpO_2_) (**A**) and cerebral oxygenation (**B**) during the cognitive task. ^†††^ *p* < 0.001 significant main effect of condition (normoxia vs. hypoxia). ^###^ *p* < 0.001 vs. rest under hypoxia. Note that cerebral oxygenation is expressed as a relative change from baseline.

Figure 4 shows SpO_2_ (A) and cerebral oxygenation (B) during the cognitive tasks. Hypoxia decreased SpO_2_ (F_1,12_ = 656.61, *p* < 0.001) and cerebral oxygenation (F_1,12_ = 238.26, *p* < 0.001) compared with normoxia. In the hypoxic condition, SpO_2_ (t_12_ = 13.05, *p* < 0.001) and cerebral oxygenation (t_12_ = 12.63, *p* < 0.001) further decreased during exercise compared with rest. In the normoxic condition, exercise did not affect SpO_2_ (t_12_ = 1.58, *p* = 0.14) or cerebral oxygenation (t_12_ = −0.83, *p* = 0.42).

**Figure 5:**
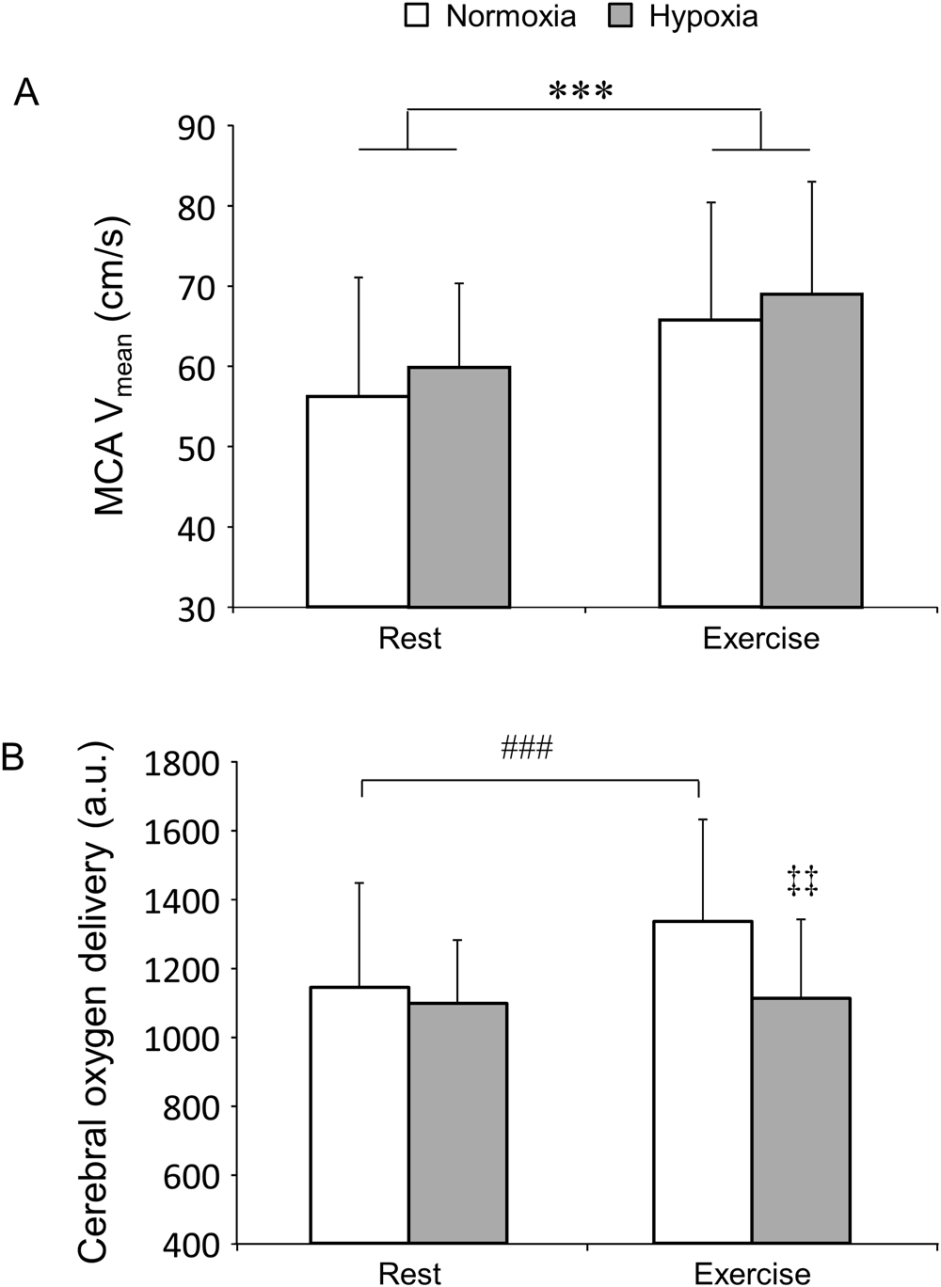
Middle cerebral artery mean velocity (MCA V_mean_) (**A**) and cerebral oxygen delivery (**B**) during the cognitive task. ^***^ *p* < 0.001 significant main effect of exercise (rest vs. exercise). ^###^ *p* < 0.001 vs. rest under normoxia. ^‡‡^ *p* < 0.01 vs. exercise under normoxia.

Figure 5 illustrates MCA V_mean_ (A) and cerebral oxygen delivery (B) during the cognitive tasks. In the present study, MCA V_mean_ increased under hypoxia compared with normoxia (rest: 10.2 ± 18.9%; exercise: 7.1 ± 16.7%). However, the increases in MCA V_mean_ under hypoxia failed to reach statistical significance, possibly due to large inter-individual differences (F_1,9_ = 2.22, *p* = 0.17). MCA V_mean_ increased during exercise compared with rest (normoxia: 18.1 ± 8.1%; hypoxia: 15.0 ± 7.8%) (F_1,9_ = 84.18, *p* < 0.001). The magnitude of MCA V_mean_ increase was no different between conditions (F_1,9_ = 0.03, *p* = 0.88). Cerebral oxygen delivery was no different between normoxia and hypoxia at rest (t_9_ = 0.83, *p* = 0.43). However, alterations in cerebral oxygen delivery differed between normoxia and hypoxia (F_1,9_ = 15.88, *p* = 0.003). Although cerebral oxygen delivery significantly increased during exercise compared with rest in the normoxic condition (t_9_ = −7.03, *p* < 0.001), it did not change in the hypoxic condition (t_9_ = −0.56, *p* = 0.59).

### Correlations analysis

**Figure 6:**
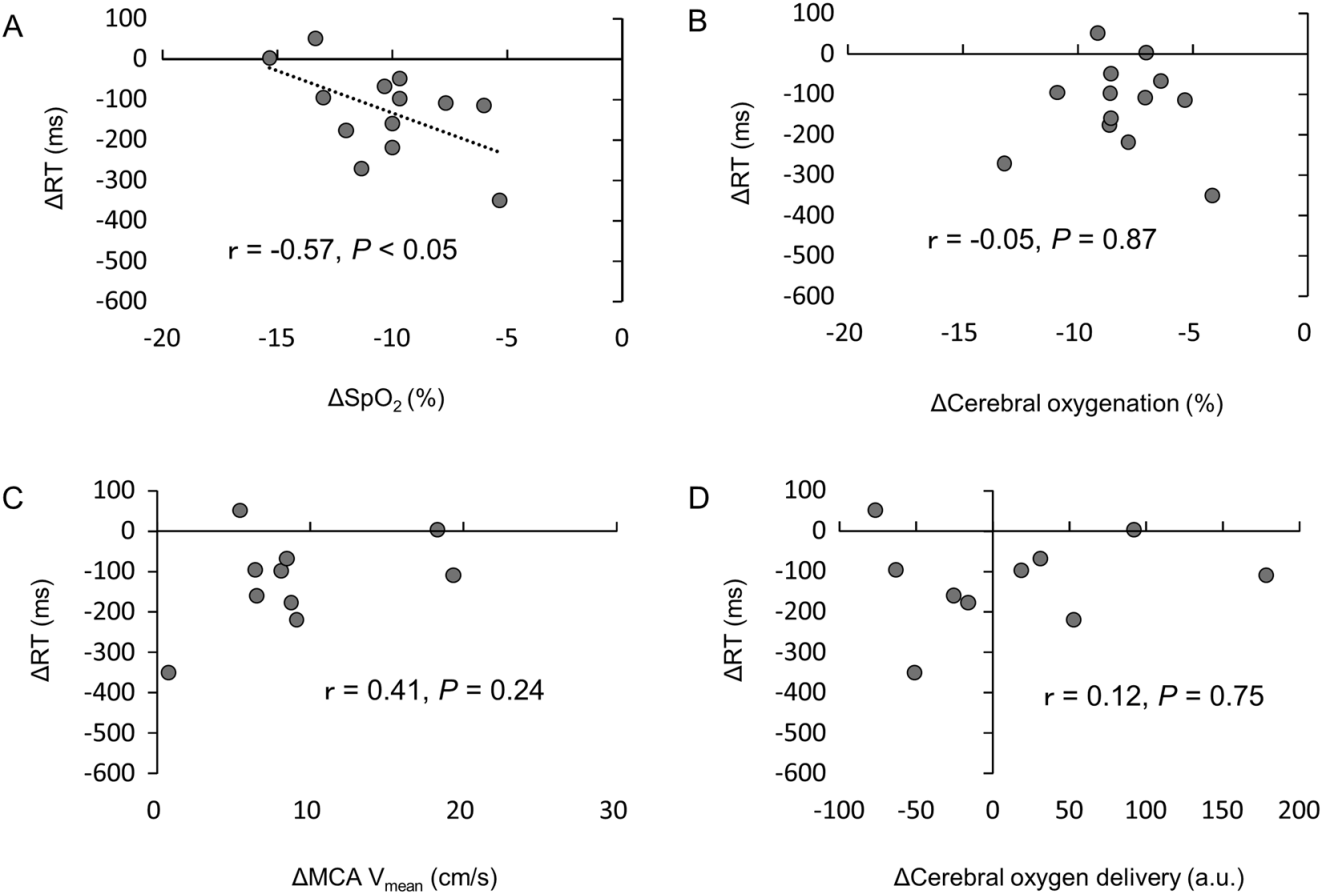
Correlation analysis between alterations in RT (∆RT) and arterial oxygen saturation (∆SpO_2_) (**A**), cerebral oxygenation (∆cerebral oxygenation) (**B**), middle cerebral artery mean velocity (∆MCA V_mean_) (**C**) and cerebral oxygen delivery (∆cerebral oxygen delivery) (**D**) under hypoxia.

In the normoxic condition, ∆RT was not associated with ∆SpO_2_ (r = 0.09, *p* = 0.76), ∆cerebral oxygenation (r = −0.12, *p* = 0.71), ∆MCA V_mean_ (r = 0.05, *p* = 0.89) or ∆cerebral oxygen delivery (r = 0.13, *p* = 0.70). In contrast, in the hypoxic condition, ∆RT was negatively correlated with ∆SpO_2_ (Fig. 6, r = −0.57, *p* = 0.043), whereas ∆RT was not associated with ∆cerebral oxygenation (r = −0.05, *p* = 0.87), ∆MCA V_mean_ (r = 0.41, *p* = 0.24) or ∆cerebral oxygen delivery (r = 0.12, *p* = 0.75).

## Discussion

The present study examined the combined effects of moderate exercise and severe hypoxia on cognitive function. The main findings of this study were: (1) severe hypoxia did not impair cognitive function at rest; (2) RT in the GNG task decreased during moderate exercise under normoxia and severe hypoxia; (3) under severe hypoxia, ∆RT was negatively correlated with ∆SpO_2_, while ∆RT was not associated with ∆cerebral oxygenation, ∆MCA V_mean_ or ∆cerebral oxygen delivery_._ These results suggest that a short bout of moderate exercise improves cognitive function, even under severe hypoxia. However, the improvements in cognitive function were attenuated in individuals exhibiting greater decreases in SpO_2_ during exercise under severe hypoxia. The present results suggest that arterial oxygen saturation attenuates cognitive improvements during exercise.

A number of previous studies have suggested that hypoxia has detrimental effects on cognitive function, and brain desaturation and resultant biological processes are thought to lead to cognitive impairments^7–9^. In the present study, at rest under hypoxia, SpO_2_ was reduced to 87.8 ± 1.5%, and cerebral oxygenation decreased by 8.9 ± 1.7%. Nevertheless, cognitive function was not impaired compared with normoxia. This result suggests that severe hypoxia and physiological alterations were not sufficient to impair cognitive function under the present experimental conditions. In the resting condition, it has been reported that elevated cerebral blood flow serves to maintain cerebral metabolism under severe hypoxia^2^. Although increases in cerebral blood flow at rest under severe hypoxia failed to reach statistical significance in the present study, cerebral oxygen delivery was maintained at rest under severe hypoxia due to increases in cerebral blood flow. This finding suggests that preserved cerebral metabolism might have contributed to the prevention of cognitive impairment at rest in the present study.

It could be argued that the present results are inconsistent with previous findings showing that hypoxia generally impaired cognitive function at rest in narrative reivews^7–9^. However, there are two possible explanations for the absence of cognitive impairment at rest in the present study. First, the cognitive task at rest was performed 10 min after the start of exposure to hypoxia, and the duration of exposure was relatively short compared with a previous study reporting that cognitive function was impaired at rest under severe hypoxia^21^. To our knowledge, it is currently unclear to what extent duration of exposure to hypoxia is related to impairments in cognitive function. However, given that physiological responses in the brain (e.g., brain swelling) occur as the duration of exposure to severe hypoxia is prolonged^24^, brain function may also be affected by duration of exposure to severe hypoxia. Therefore, in the present study, although cognitive function was performed after SpO_2_ became stable in all participants, the absence of the impairments in cognitive function at rest may be related to the relatively short duration of exposure to severe hypoxia.

Another possible explanation for the absence of impairments in the current findings is task specific effects of hypoxia and/or task difficulty. It has been suggested that some domains may not be sensitive to acute hypoxia^25, 26^. Furthermore, in a previous study, executive function and reaction time were not impaired under severe hypoxia while complex attention, composite memory, and psychomotor speed were impaired^27^. These findings suggest that executive function could be robust to severe hypoxia. Hence, the absence of cognitive impairments may be associated with the cognitive tasks used in the present study. Next, the present cognitive task was a modified version of multitasking. Hence, several brain areas, including the prefrontal and parietal cortex, are likely to have been activated when participants performed the task^19^. Nevertheless, high accuracy of cognitive performance was maintained throughout the experiment. This suggests that the task difficulty was relatively low once the participants were familiar with the task. Given that task difficulty is one of the key factors influencing exercise-cognitive function^28, 29^, it is possible that the difficulty of the present task was not high enough to evoke impaired cognitive function at rest under severe hypoxia. Collectively, a second possible explanation for the absence of cognitive impairments may be task specific effects of hypoxia and/or task difficulty.

In the present study, we examined how moderate exercise affects cognitive function under severe hypoxia. During exercise under hypoxia, SpO_2_ was reduced to 77.5 ± 3.0%, and cerebral oxygenation decreased by 17.0 ± 2.4%. Decreased cerebral oxygenation is typically caused by a decrease in oxyhaemoglobin (oxy-Hb) and a reciprocal increase in deoxyhaemoglobin (deoxy-Hb). Nevertheless, moderate exercise improved cognitive function under severe hypoxia as well as normoxia. Furthermore, the magnitude of improvement in cognitive function was no different between normoxia and severe hypoxia. These results suggest that, on average, the beneficial effects of moderate exercise on cognitive function are still intact even under severe hypoxia.

In the present study, RT decreased during moderate exercise under normoxia and severe hypoxia. However, neither exercise nor hypoxia affected the accuracy of the cognitive task. These results indicate that decreases in RT were not related to a speed-accuracy trade-off. Rather, we can assume that the observed decreases in RT were primarily due to physiological alterations induced by acute exercise. Although the precise physiological mechanisms are currently unclear, increases in arousal level are thought to be associated with improvements in cognitive function during exercise^11, 13^. Exercise affects brain circuits involving a number of neurotransmitters, including dopamine, noradrenaline, serotonin, adrenocorticotropic hormone, and cortisol^14, 30^. These physiological alterations might be involved in modulating neural activity, possibly leading to improvements in cognitive function^16^. Importantly, exercise has been found to facilitate implicit information by enhanced noradrenergic and dopaminergic systems^14^. Thus, it is possible that the noradrenergic and dopaminergic systems play a key role in improving cognitive function^15, 16^.

We observed that RT decreased during moderate exercise under severe hypoxia, and ∆RT was negatively correlated with ∆SpO_2_ under severe hypoxia. In contrast, ∆RT was not correlated with ∆cerebral oxygenation, ∆MCA V_mean_ or ∆cerebral oxygen delivery. Thus, the present results suggest that decreases in SpO_2_ potentially attenuate cognitive improvements during exercise hypoxia, supporting the notion that PaO_2_ is the key predictor of cognitive function under hypoxia in the resting condition^10^. The present findings seem to extend the notion to exercise under hypoxia. At the cellular level, the turnover of several neurotransmitters appears to be altered under hypoxia^31^. Hence, alterations in the turnover of neurotransmitters may attenuate cognitive improvements during exercise under severe hypoxia.

Cerebral oxygenation reflects the balance between oxygen availability and utilisation^32^. A decrease in cerebral oxygenation suggests that oxygen availability may not be sufficient to meet the metabolic demands of the brain. However, in contrast to our hypothesis, ∆RT was not associated with ∆cerebral oxygenation in the present study. This suggests that, under the present experimental conditions, alterations in cerebral oxygenation detected by near-infrared spectroscopy (NIRS) may not provide sufficient resolution to identify the link between cognitive function and prefrontal oxygenation. Cerebral oxygenation measured by NIRS reflects the combined oxygenation of an uncertain mixture of the arterioles, capillaries, and post-capillary veins, and the venous contribution to cerebral oxygenation is far greater than the arterial contribution^33^. Thus, greater venous contribution to cerebral oxygenation may account for the absence of the association between ∆RT and ∆cerebral oxygenation in the current study. This would in turn highlight the importance of arterial oxygen saturation and resultant biological processes for cognitive function during exercise under severe hypoxia.

In the present study, ∆RT was not associated with ∆MCA V_mean_. This indicates that alterations in cerebral blood flow were not directly associated with alterations in cognitive function during exercise under hypoxia. The present results also suggest that improvements in cognitive function during exercise were not directly associated with increases in MCA V_mean_ under normoxia, in line with previous reports^34, 35^. In terms of cognitive function, alterations in cerebral blood flow may not be matched by changes in oxygen delivery to the brain or by increased cerebral metabolism^35^. However, given that cerebral blood flow contributes to the increases in oxygen delivery to the brain during exercise, this does not mean that increased cerebral blood flow is unnecessary for improving cognitive function. During exercise, cerebral oxygen delivery was lower under hypoxia compared with normoxia. The brain increases non-oxidative metabolism during exercise under severe hypoxia^3^. Hence, increased non-oxidative metabolism could help maintain cerebral metabolism, which may account for the absence of an association between ∆RT and ∆cerebral oxygen delivery.

The present results suggest that arterial oxygen saturation may play a key role in the attenuation of cognitive improvements during exercise under hypoxia. Nevertheless, ΔRT varied widely among participants and it is unclear why there was such a large variation. Hence, we do not assume that decreases in SpO_2_ is the sole factor involved in the detrimental effects on cognitive function during exercise under severe hypoxia. Together with physiological effects examined in the present study, exercise under hypoxia has other physiological effects on the human brain including metabolism, neuronal excitability, and oxidative stress^2, 6, 36^. Hence, the effects of acute exercise under hypoxia on cognitive function are likely to be determined by the integration of multiple factors. In addition, given that none of the cortical measures showed a correlation with ΔRT, we cannot rule out the possibility that the negative correlation might, at least in part, reflect associations between cognitive performance and other organs (e.g. skeletal muscle). Future studies will be required to elucidate the specific mechanisms by which acute exercise affects cognitive function under hypoxia.

One may argue that RT in the present GNG task was longer as compared with the previous studies^37–40^. We think that there are two reasons to account for the longer RT. First, in the present study, paired figures were relatively similar in shape so that task difficulty and cognitive demands were presumably high (see example in Fig. 2). Thus, participants required relatively long time to discriminate the figures. Second, participants performed the GNG task while remembering the location of the visual stimulus in the spatial DR task. This required working memory and potentially made reaction time longer. Indeed, our previous study using only the GNG task^17^ exhibited shorter RT as compared with RT in the GNG task when both GNG and spatial DR tasks were combined^19, 41^. Therefore, although RT in the present cognitive task was relatively long, we can assume that the present results are valid.

In the present study, the durations of exposure to hypoxia and exercise were relatively short. This may have limited the combined effects of severe hypoxia and exercise on cognitive function. The combined effects of prolonged exposure to hypoxia and/or prolonged exercise should be further investigated. Second, absolute workload was much lower under hypoxic conditions compared with normoxic conditions. Thus, it is possible that differences in absolute exercise intensity affected the present results. Further studies with the same absolute exercise intensity between normoxia and hypoxia conditions may be useful for clarifying the combined effects of hypoxia and exercise on cognitive function. Third, the number of participants may also be a limitation in the present study. We cannot rule out the possibility that the small number of the participants could at least in part affect the correlation analysis. Finally, we used two fraction of inspired oxygen (FIO_2_) levels (0.12 and 0.13), which could be a methodological limitation. In the future studies, it is necessary to examine cognitive function during exercise under hypoxia at various FIO_2_ levels in a graded manner.

The diameter of the MCA remains constant under different physiological conditions^42^. However, the MCA has been found to dilate under severe hypoxia^43^. Moreover, recent studies have suggested that transcranial Doppler ultrasonography (TCD), as a measure of cerebral blood flow, underestimates volumetric changes^44^. Hence, it is possible that the alterations in cerebral blood flow velocity under severe hypoxia observed in the current study did not directly reflect variations in cerebral blood flow. Furthermore, we measured only MCA V_mean_, and the distribution of cerebral blood flow was not determined. Given that blood flow in response to hypoxia appears to differ between the brainstem and the cortex^45^, further studies are required to clarify how alterations in regional cerebral blood flow and cerebral oxygen delivery affect cognitive function during exercise under hypoxia.

In summary, we examined the combined effects of a single bout of moderate exercise and severe hypoxia on cognitive function. Cognitive function improved during a short bout of exercise under severe hypoxia, suggesting that the beneficial effects of exercise on cognitive function persisted under severe hypoxia. Nevertheless, the improvements were attenuated in individuals exhibiting a greater decrease in arterial oxygen saturation. Hence, the current findings suggest that arterial desaturation attenuates improvements in cognitive function during exercise.

## Materials and Methods

### Participants

Thirteen male participants (age = 21.5 ± 3.5 years; height = 1.73 ± 0.05 m; body mass = 66.4 ± 10.6 kg) took part in this study. Participants were physically active and did not have any history of cardiovascular, cerebrovascular, or respiratory diseases. All participants provided written informed consent. This study was approved by the Human Research Committee of the Research Centre of Health, Physical Fitness and Sports, Nagoya University and was in accordance with the Declaration of Helsinki.

### Experimental procedure

The experiment consisted of five sessions. In the first two sessions, participants performed maximal exercise tests using a cycle ergometer (Aerobike 75XLIII; Combi, Tokyo, Japan) while breathing either a normoxic (FIO_2_ = 0.209) or hypoxic gas mixture (FIO_2_ = 0.12 or 0.13), in a single-blind randomised design. The hypoxic gas corresponds to altitudes of approximately 3,800 or 4,500 m. Normoxic and hypoxic gases were provided by a gas generator^46^ through a facemask. The lower limit for SpO_2_ during the maximal exercise test in hypoxia was set at 70%. Seven participants exhibited SpO_2_ values below 70% during exercise while breathing a 12.0% gas mixture. Therefore, we used a 13.0% O_2_ gas mixture for these participants in the hypoxia condition. This procedure was aimed to reduce the variability of SpO_2_ during exercise under hypoxia and to prevent further decrease in SpO_2_. Indeed, SpO_2_ was 77.5 ± 3.0% during exercise under hypoxia and fell within the target range. After the wash-in period for 10 min, the maximal exercise test started at an initial power output of 90 watts (W), and the workload was increased by 15 W/min until exhaustion^46^. The participants maintained a cycling cadence of 60 rpm, and the test was stopped when participants were not able to maintain a cadence > 55 rpm for five consecutive revolutions. During the maximal exercise tests, the peak ${\dot{{\rm{V}}}{\rm{O}}}_{{\rm{2}}}$ $({\dot{{\rm{V}}}{\rm{O}}}_{{\rm{2peak}}})$ was taken as the highest ${\dot{{\rm{V}}}{\rm{O}}}_{{\rm{2}}}$ measured, after which workloads at 30% and 50% ${\dot{{\rm{V}}}{\rm{O}}}_{{\rm{2peak}}}$ were calculated under normoxia and hypoxia. Thus, relative exercise intensities were the same between the normoxic and hypoxic conditions. In the third session, participants completed three practice blocks of the cognitive tasks (both spatial DR and GNG tasks) at rest and during cycling until all RT in the Go trial fall within three SD from the mean of 30 trial. When RTs did not fall within three SD, the participants continued practice blocks until they met the criteria. We expected that this familiarization period would minimize learning effects. In the remaining sessions (Fig. 1), participants performed the cognitive task at rest and during exercise under either normoxia or normobaric hypoxia in a random order. Participants were blinded to the respective condition. In the hypoxic condition, we used the same concentration (FIO_2_ = 0.12 or 0.13) for each participant as in the maximal exercise test. First, participants were exposed to the normoxic or hypoxic gas for at least 10 min, followed by the cognitive task on the cycle ergometer. One minute after the first cognitive task, the participants performed cycling on the ergometer. Following exercise at 30% ${\dot{{\rm{V}}}{\rm{O}}}_{{\rm{2peak}}}$ for 5 min as a warm-up, participants cycled at 50% ${\dot{{\rm{V}}}{\rm{O}}}_{{\rm{2peak}}}$ for 15 min. The second cognitive task was started 5 min after exercise intensity reached 50% ${\dot{{\rm{V}}}{\rm{O}}}_{{\rm{2peak}}}$.

### Cognitive tasks

In the present study, we used two cognitive tasks (the spatial DR task and the GNG task) that require executive function^19, 41, 47^. We assessed executive function because it includes basic cognitive processes such as inhibition, working memory, and cognitive flexibility^48^. The present cognitive task was initiated by appearing the stimulus of spatial DR task (Fig. 2). The visual stimulus was presented in one of eight locations surrounding a fixation point. Participants were instructed to memorise the location at which the visual stimulus was presented. Next, the GNG task was conducted. On each trial, one of a pair of figures was presented at the centre of the computer display. At the beginning of the GNG task, the participants did not know which figure corresponds to the Go trial. They identified the relationship between correct response and the figure from a correct or incorrect feedback tone. On any given trial, if the presented figure was the target (“Go trial”), participants released a shift key as quickly as possible. If the figure was not the target (“No-Go trial”), participants continued holding the shift key down. After a trial in the GNG task, participants were required to recall the location at which the visual stimulus was presented in the preceding spatial DR task. Visual stimuli were presented at eight locations surrounding the fixation point. The participants pressed the button on a portable 10-key pad to indicate the location they remembered. The sequence was defined as a trial.

The portable 10-key pad and computer keyboards were horizontally positioned above both sides of the ergometer’s handlebars. The participants pressed the 10-key pad with their right index finger (spatial DR task) and pressed the shift button on the keyboard with their left index finger (GNG task). After participants had completed five or six successive trials in the GNG task, the other figure became the target. After the next five or six successive trials were completed, a new pair of figures was presented. We used different pattern of changing the trial during successive trials. The participants did not know when the correct response and the figure would be reversed, or when the new pair of figures would be presented. The cognitive tasks continued until participants had completed 30 trials of each task. In the GNG task, Go and No-Go trials were randomly presented, and actual percentage of Go trial was 49.2 ± 4.6%. The average time to complete the cognitive tasks was 308 ± 24 s. Despite some arguments that inhibitory requirements are greatly reduced in the GNG task^40^, the present cognitive tasks are thought to require executive function.

Error trials in the spatial DR task were incorrect responses to the remembered location. Error trials in the GNG task were defined as omitting a response in a Go-trial, or performing an incorrect response in a No-Go trial. Accuracy in the respective tasks was calculated as the number of correct trials divided by the total number of trials. RT was calculated for correct trials as the time between stimulus presentation and response. To assess the GNG task performance, we excluded trials occurring immediately after reversal of the relationship between correct responses and figures, and trials immediately after presentation of a new pair of figures.

### Measurements

SpO_2_ was monitored using a pulse oximeter (Biox 3740; Datex-Ohmeda Inc., Madison, WI, USA) placed on the left middle finger. Cerebral oxygenation was continuously monitored with temporal resolved NIRS (BOM-L1 TRW; Omegawave, Tokyo, Japan)^17^. A probe holder was attached at the left side of the forehead. The source generated three wavelengths of near-infrared light (780, 810, and 830 nm). The modified Beer–Lambert law enables the continuous measurement of concentration changes in oxy-Hb and deoxy-Hb^49^. Total haemoglobin (total-Hb) is the sum of oxy-Hb and deoxy-Hb. Cerebral oxygenation is expressed as oxy-Hb/total-Hb × 100. We measured cerebral oxygenation for 30 s as a baseline while the participants were at rest under normoxia. Cerebral oxygenation during the cognitive task was expressed relative to the baseline. MCA V_mean_ was monitored using 2-MHz transcranial Doppler ultrasonography (TCD-X; Atys Medical, Rhone, France). The right MCA was insonated from the temporal region of the skull. The position and angle of the probe were adjusted for each participant, and the probe was fixed with an adjustable headband and adhesive ultrasonic gel. Arterial oxygen content (CaO_2_) was estimated using the following equation^50^: haemoglobin concentration [Hb] × 1.36 × SpO_2_/100. We assumed [Hb] was 15.0 g/dl^51^. We calculated cerebral oxygen delivery as the product of CaO_2_ × MCA V_mean_^50^.

During the cognitive task, we recorded ${\dot{{\rm{V}}}}_{{\rm{E}}}$ and P_ET_CO_2_. ${\dot{{\rm{V}}}}_{{\rm{E}}}$ was determined using an online system with a mixing chamber^46^. For the measurement of P_ET_CO_2_, sample gas was drawn through a sampling tube connected to a mask. Fraction of end-tidal CO_2_ (F_ET_CO_2_) was analysed using a gas analyser (MG-360, Minato Ikagaku, Osaka, Japan), and P_ET_CO_2_ was calculated from data of F_ET_CO_2_. A three-lead electrocardiogram (ECG) was set up, and HR was calculated from the intervals between the R waves. Arterial blood pressure (BP) was measured from the right arm at rest and during exercise using an automated BP unit (STBP-780; Colin Medical Instruments, San Antonio, TX, USA). The right forearm was placed on an arm rest to keep the limb steady and relaxed while arterial BP was measured. The sound signal was synchronised to the ECG-R wave, and a detection algorithm was used to determine the SBP and DBP. Participants reported their RPE (6–20 Borg scale)^52^ immediately after each cognitive task. Blood lactate and glucose levels were measured at rest and immediately after exercise. The right earlobe was pricked with a lancet and 2 µL of capillary blood was collected. Blood lactate concentration was determined using the lactate oxidase method (Lactate Pro 2 LT-1730; Arkray, Kyoto, Japan). Blood glucose level was measured using the glucose oxidase method (Glutest Ace; Sanwa Kagaku, Nagoya, Japan).

### Statistical analysis

We averaged SpO_2_, cerebral oxygenation, and HR during the cognitive task. ${\dot{{\rm{V}}}}_{{\rm{E}}}$, P_ET_CO_2_, and MCA V_mean_ were averaged over the last 1 min of the cognitive task. Since we did not obtain reliable images of the MCA from two participants in the normoxic condition and three participants in the hypoxic condition, data from 10 participants were included in the ANOVA in the final analysis. For correlation analysis, we used data from 11 participants (normoxia) and 10 participants (hypoxia). We determined effect size (0.5) from our previous results of RT in the Go trial^19^ because RT in the Go trial was the primary outcome. When effect size was set at 0.5, nine participants would be needed to detect a significant difference in a two-way repeated-measures ANOVA with a power of 80% and a significance level of 5%. Hence, the sample size was adequate in the present study. We performed paired t-tests for comparison of ${\dot{{\rm{V}}}{\rm{O}}}_{{\rm{2peak}}}$ at exhaustion, absolute workload at 50% ${\dot{{\rm{V}}}{\rm{O}}}_{{\rm{2peak}}}$, and actual percentage of ${\dot{{\rm{V}}}{\rm{O}}}_{{\rm{2peak}}}$ during exercise at 50% ${\dot{{\rm{V}}}{\rm{O}}}_{{\rm{2peak}}}$ between normoxia and hypoxia. A two-way repeated-measures ANOVA was performed with Condition and Exercise as within-subject factors. When an interaction was observed, we performed a paired t-test with Bonferroni correction. The Kolmogorov–Smirnov test indicated non-normal distribution only in the accuracy of the cognitive task. However, the Friedman test indicated that neither Condition nor Exercise affected the accuracy of the cognitive task (all *p*-values > 0.25). Thus, further analysis was not conducted. In the present study, as noted above, we excluded trials occurring immediately after reversal of the relationship between correct responses and figures, and trials immediately after presentation of a new pair of figures. After these trials were excluded, we compared mean RT in the Go trial with RT in the first Go trial after the participants were aware that the relationship between correct responses and figures had been changed. This was aimed to examine whether the participants adapted to new relationships immediately. For the analysis, we performed paired t-tests in four conditions (rest under normoxia, exercise under normoxia, rest under hypoxia, and exercise under hypoxia) with Bonferroni correction. Pearson’s correlation coefficient was calculated to establish a correlation between alterations in RT (∆RT, ms) and ∆physiological variables. ∆RT and ∆physiological variables were calculated by subtracting respective values at rest from those during exercise. All data are expressed as mean ± SD. The significance level was set at *p* < 0.05.
